# Biochemical Frequency Control by Synchronisation of Coupled Repressilators: An *In Silico* Study of Modules for Circadian Clock Systems

**DOI:** 10.1155/2011/262189

**Published:** 2011-10-20

**Authors:** Thomas Hinze, Mathias Schumann, Christian Bodenstein, Ines Heiland, Stefan Schuster

**Affiliations:** Department of Bioinformatics, Faculty of Biology and Pharmacy, Friedrich Schiller University Jena, E.-Abbe-Platz 1-4, 07743 Jena, Germany

## Abstract

Exploration of chronobiological systems emerges as a growing research field within bioinformatics focusing on various applications in medicine, agriculture, and material sciences. From a systems biological perspective, the question arises whether biological control systems for regulation of oscillatory signals and their technical counterparts utilise similar mechanisms. If so, modelling approaches and parameterisation adopted from building blocks can help to identify general components for frequency control in circadian clocks along with gaining insight into mechanisms of clock synchronisation to external stimuli like the daily rhythm of sunlight and darkness. Phase-locked loops could be an interesting candidate in this context. Both, biology and engineering, can benefit from a unified view resulting from systems modularisation. In a first experimental study, we analyse a model of coupled repressilators. We demonstrate its ability to synchronise clock signals in a monofrequential manner. Several oscillators initially deviate in phase difference and frequency with respect to explicit reaction and diffusion rates. Accordingly, the duration of the synchronisation process depends on dedicated reaction and diffusion parameters whose settings still lack to be sufficiently captured analytically.

## 1. Introduction

In both spheres, biological and technical systems, oscillatory signals play a major role in order to trigger and control time-dependent processes. Core oscillators are the simplest devices for generation of continuously running clock signals. To this end, signal processing units consisting of at least one feedback loop can suffice [1]. So, it is no surprise that probably numerous evolutionary origins led to oscillative reaction networks while independently technical attempts succeeded in construction of single clocks or clock generators.

The situation becomes more complicated if several of those core oscillators start to interact. Resulting biological systems are commonly driven to achieve a synchronous behaviour towards an evolutionary advantage. Correspondingly, clock synchronisation in technical systems is frequently inspired by the need to follow a global time. Interestingly, the formalisation of clock synchronisation processes is quite distant from each other. While, in distributed computer systems, stepwise algorithmic approaches (like Berkeley or Christian's method, [2]) predominate, biological systems adjust their clock signals more gradually, which might include sequences (cascades) of dedicated modifications in spatial molecular structures or even consideration of compartmental dynamics. Typically, its process formalisation is either based on reaction-diffusion kinetics or employs more abstract analytic techniques adopted from general systems theory whose range of application preferably covers sinusoidal signal shapes. For this purpose, the Kuramoto method [3], an analytic signal coherence measure to counteract phase shift between each pair of core oscillators, became established among others. These state-of-the-art approaches have in common to exploit systems of differential equations derived from kinetic laws of the underlying set of involved reactions as well as transportation or diffusion processes. Coping with the complexity of those monolithic models that can easily contain more than one hundred single equations and several hundred parameters to be reasonably fitted is a challenging and error-prone task. Additionally, the modelling often coincides with some incomplete, imprecise, or partially wrong information about the desired reaction network topology and its kinetic parameters. To overcome these insufficiencies in modelling, we suggest a specific concept of reaction system *modularisation* inspired by engineering.

Our concept is based on the assumption that *“structure follows function.”* Although there is a plethora of different strategies and implementations to achieve a certain complex network function, the pool of sufficient network candidates can be divided into compositions of a low number of elementary functional units called *modules*. This term is not new in systems biology when considering recurrent motifs conserved in metabolic, cell signalling, and gene regulatory networks. We extend the notion of modules in terms of information processing. In this context, a module is able to fulfill an elementary *computational task*. Here, the spatiotemporal course of substrate concentrations along with molecular and compartmental structures acts as data carrier. Beyond logic and arithmetic functions carried out by the module's steady-state behaviour, simple buffer and delay elements contribute to a collection of biochemical modules, each of which comprising few molecular species and a maximum number of reactions within the same magnitude. When combining those modules towards reaction networks capable of a more complex functionality, we permit so-called *shared molecular species* among distinct modules. This way of module coupling enables compact network topologies in accordance with evident observations from *in-vivo* studies. Moreover, there is no need for further separate interface specifications. In most cases, the behaviour of a module can be captured by chemical counterparts of *transfer functions* in conjunction with characteristic curves which exhibit an established practice in engineering. Utilisation of transfer functions for modules significantly reduces the number of distinct parameters to be considered by keeping the relevant characteristics of the entire network.

Within a case study, we exemplify oscillatory signal synchronisation by a biological system composed of bidirectionally coupled repressilators. To this end, we model the entire gene regulatory networks using reaction-diffusion kinetics. Afterwards, we conduct two comprehensive simulation studies. The first one discloses the time to synchronisation subject to initial phase shift between the elementary repressilators. Its balanced diffusion rate acts as coupling strength. It appears that synchronisation of initially antiphasic signals is most time consuming for weak coupling while it has a negligible effect for strong coupling. A second simulation study investigates the synchronisation behaviour with respect to different initial frequencies of the single repressilators.

Coupled repressilators represent a prototypic example of core oscillators embedded into a complex reaction-diffusion network constituting a circadian clock as an entire system able to be adjusted by external stimuli. From a systems point of view, circadian clocks form biochemical frequency control circuits whose functionality resembles technical counterparts utilising so-called *phase-locked loops*. Corresponding circuits comprise three essential modules:

- a core oscillator whose frequency has been controlled to adapt to an external stimulus,
- a signal comparator (phase detector) responsible for determining the deviation between the signal produced by the core oscillator on the one hand and the external stimulus on the other one,
- a biochemical low-pass filter.

Finally, we illustrate a corresponding scheme by the identification of feasible network candidates (modules) composing a pure biochemical frequency control by a phase-locked loop.

Beyond frequency control circuits, there are some examples on how biological and technical systems utilise equal or similar mechanisms to achieve a certain function. In the context of fascinating approaches within biomimicry [4], those relationships become more and more obvious. Even at a nanoscopic scale, we can detect homologies between electronic circuits and molecular reaction systems. For instance, biobricks [5] can act as logic gates while cell signaling motifs comprise the function of signal amplifiers, buffers, or filters like low-pass filters [6]. All these components contribute to astonishing capabilities of biological information processing. Biochemical clockworks by controllable oscillatory signals and their technical counterparts, frequency control by phase-locked loops, can support the idea of similar information processing techniques employed in nature and in engineering. Nevertheless, the pool of potential problem solutions in nature seems to be currently much larger than in recent engineering, which motivates the progression of interdisciplinary research in systems biology.

## 2. Prerequisites

We define different temporally oscillating signals to be *synchronous* to each other if and only if they meet three conditions.

1. The oscillatory signal must run *undamped* to avoid signal weakening.
2. *Asymptotical or total harmonisation* of the oscillatory signals meaning that after a finite amount of time called *t*_sync_ (time to synchronisation), both temporal signal courses converge within an arbitrarily small *ε*-neighbourhood.
3. The resulting oscillatory signal after *t*_sync_ has to be *monofrequential* to ensure chronoscopy (constant progression of time measure).

Note that synchronicity is stricter than obtaining entrainment. While an adaptation of oscillatory signal frequency and phase suffices for entrainment, synchronicity additionally requires harmonisation of the entire signal shape (waveform) over time.

The central prerequisite of a core oscillator to be capable of synchronisation to others is its ability to vary its oscillation frequency within a specified range [7]. This variation can be achieved by *forcing*, by *resetting*, or by specific *selective perturbations* affecting the oscillating signal. Without any external influences, core oscillators resume their individual free-running oscillatory behaviour, mostly by loosing their synchronicity. 

Topologically, clock synchronisation can be accomplished by two different strategies called *external* and *internal* [8]. External strategies comprise a central leading clock that propagates its time signal throughout the whole network of downstream core oscillators which adjust their individual signals by accelerating or slowing down their frequency for a certain amount of time. Here, we observe a unidirectional coupling from the leading central clock to all others. In contrast, internal strategies aim at a mutual clock exchange between the network members. The coupling topology is mostly bidirectional, and each involved core oscillator is going to adjust its signal based on a weighted sum of the signals released by its adjacent clocks.

## 3. The Repressilator: A Goodwin-Type Controllable Oscillator

There are numerous biochemical core oscillators found in living organism's clocks [9]. From today's perspective, its majority reveals the Goodwin type [10]. A Goodwin-type oscillator comprises an abstract metamodel of a cyclic gene regulatory network, which is able to exhibit a sustained oscillatory behaviour in its substrate concentrations. Goodwin-type oscillators have in common three dedicated focal substrates typically called *X*, *Y*, and *Z* in which *X* represents a mRNA translated into a protein *Y* within the cytoplasm. *Y* is transported into the nucleus where it functions as a repressor *Z*, which in turn inhibits the transcription of *X*. All focal components (*X*, *Y*, and *Z*) degrade in the presence of specific proteases acting as catalysts. It turns out that the velocity of degradation is the most effective way to control the oscillation frequency [11].

The original Goodwin oscillator, a prototypic core model for generation of endogenous circadian rhythms, comes with an attribute worth to be revised towards a more biochemical model. The inhibition of X utilises a Hill term (derived from saturation kinetics, [12]) whose Hill coefficient demands an unrealistically high value of 9 or higher to ensure sustained oscillations. Since the Hill coefficient typically coincides with the number of reactive binding sites assigned to the repressor protein Z, one would normally expect values below or equal to 4. 

Nevertheless, the Goodwin model became established in terms of a general formalism (building block module) to capture the dynamical behaviour of circadian clock systems based on ordinary differential equations. Particularly in the beginning of chronobiological research, the Goodwin model was useful to act as a placeholder for partially unknown reaction network topologies. Later, it turned out that circadian clocks residing in manifold life forms resemble the Goodwin approach in principle, but not exactly [9]. For instance, the circadian core oscillator found in *Arabidopsis thaliana* (a flowering plant within the family of mustards, *Brassicaceae*) [13] as well as its counterparts in some mammalians [14] can be described by slightly modified forms of the Goodwin model. Even the core components of the human circadian clock located in the suprachiasmatic nucleus are mainly in accordance with the oscillatory mechanism of the Goodwin model [15].

In each organism, the biochemical core oscillator or a corresponding system of coupled core oscillators is embedded into an entire clockwork that ensures additional features beyond a simple oscillatory behaviour like its ability to entrainment or its capability of temperature compensation within a physiological range. Moreover, substrates involved in clock mechanisms often act as trigger species. They frequently undergo various perturbations, which affect the according species concentrations over time. In total, it appears that a core oscillator's reaction network in general is strongly interwoven with further reaction systems responsible for cell signalling or for metabolic activities. Unravelling all facets of those complex control loops encourages a strict modularization in order to separately identify the effect and the intensity of all individual stimuli involved in the entire clockwork. For a first study following this line, we are going to have a core oscillator at hand whose formal model could be successfully verified using an *in-vivo* study and whose interconnection with other reaction systems within an organism is low.

The repressilator [16] seems to be an appropriate candidate for our purposes. A repressilator is a gene regulatory network of the Goodwin type consisting of three focal proteins (LacI, TetR, and cI) that mutually inhibit their expression from genes (*lacI, tetR, cI*). Using two synthetic plasmids, a repressilator had been successfully embedded into a strain of *Escherichia coli* [16]. LacI in concert with TetR, two of the repressilator's focal proteins, is potentially able to interact with AHL (N-acyl homoserine lactone) in terms of quorum sensing [17, 18]. This implies the possibility of a mutual exchange of molecules between individual repressilators forming a bidirectional repressilator coupling. In the resulting system, it is of interest to explore its behaviour towards synchronisation of individual oscillations. Getting insight into the underlying mechanisms from a modelling perspective could elucidate the robustness of biological clockworks against perturbations. Even the human circadian clock utilises a number of bi- and unidirectionally coupled core oscillators [19]. Moreover, it is generally believed that there are several oscillators interconnected with each other sensing different time cues [20–22]. By choosing a system composed of two coupled repressilators, we can explore its inherent properties by keeping the entire system simple.

## 4. Internal Synchronisation: Coupled Repressilators

### 4.1. Reaction Network and Kinetics

**Figure 1:**
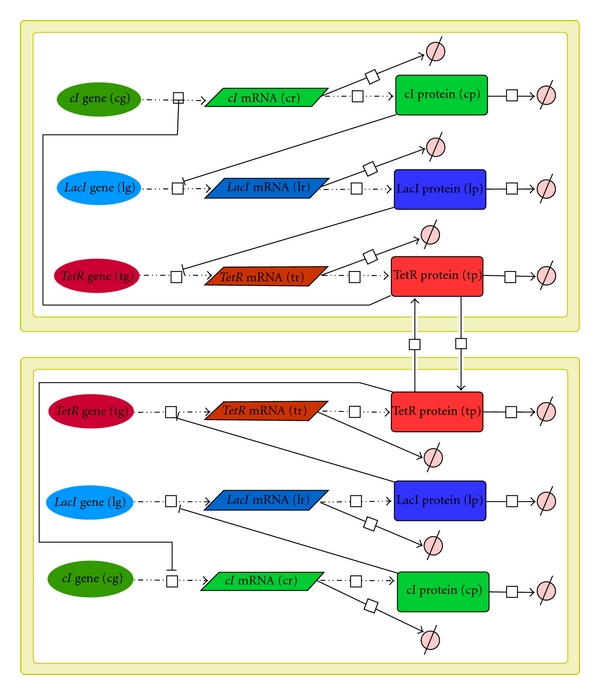
Network topology of the TetR-coupled repressilator model with diffusion between both core oscillators.

We identified a network of bidirectionally coupled repressilators to be an appropriate candidate to explore internal synchronisation within a biological system. We employ a system composed of two coupled repressilators located in two adjacent cells inspired by Garcia-Ojalvo et al. [17], see Figure 1. 

Let TetR be a protein assumed to be able to migrate between the cells, it acts as coupling element. Its diffusion rate diff specifies the variable bidirectional coupling strength. The dynamical behaviour of the network can be specified by reaction-diffusion kinetics based on corresponding ordinary differential equations (ODEs). For species names in the ODEs, we abbreviate (LacI, TetR, cI) = (*lp, tp, cp*) for the proteins and (*lacI, tetR, cI*) = (*lr, tr, cr*) for the mRNA. The set of equations for each single repressilator reads:

(1) $$\begin{array}{c} \frac{\text{d}lp}{\text{d}t}=k_{lr}\cdot lr-k_{lp}\cdot lp,\\ \frac{\text{d}tp}{\text{d}t}=k_{tr}\cdot tr-k_{tp}\cdot tp-\text{diff}\cdot tp+\text{diff}\cdot tp_{\text{external}},\\ \frac{\text{d}cp}{\text{d}t}=k_{cr}\cdot cr-k_{cp}\cdot cp,\\ \frac{\text{d}lr}{\text{d}t}=\alpha_{0}+\frac{\alpha \cdot k_{m}^{n}}{k_{m}^{n}+cp}-k_{lr}\cdot lr-k_{lr2}\cdot lr,\\ \frac{\text{d}tr}{\text{d}t}=\alpha_{0}+\frac{\alpha \cdot k_{m}^{n}}{k_{m}^{n}+lp}-k_{tr}\cdot tr-k_{tr2}\cdot tr,\\ \frac{\text{d}cr}{\text{d}t}=\alpha_{0}+\frac{\alpha \cdot k_{m}^{n}}{k_{m}^{n}+tp}-k_{cr}\cdot cr-k_{cr2}\cdot cr. \end{array}$$

We utilise the parameter setting *α*_0_ = 0.03, *α* = 29.97, *k*_*m*_ = 40, *n* = 3, *k*_{*lp*,*tp*,*cp*}_ = 0.069, *k*_{*lr*,*tr*,*cr*}_ = 6.93, and *k*_{*lr*2,*tr*2,*cr*2}_ = 0.347 resulted from a parameter fitting based on the available experimental data [17]. Additionally, the initial species concentrations in case of no phase shift are chosen at the limit cycle, for example, *lr* = 0.819, *tr* = 2.388, *cr* = 0.068, *lp* = 36.263, *tp* = 166.685, and *cp* = 64.26.

**Figure 2:**
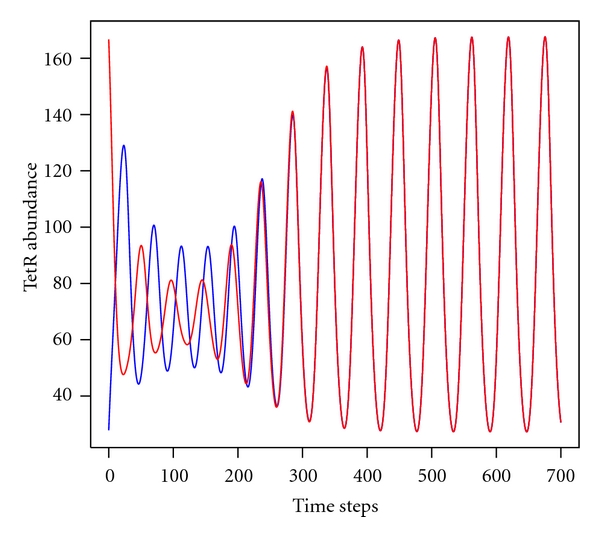
Typical synchronisation run of two coupled repressilators, coupling strength diff = 0.04, initial phase shift 182° (arbitrarily chosen). Simulation carried out with Copasi using ODEs and parameter settings given in Section 4.1.

The repressilator's oscillation frequency mainly depends on the degradation reaction rates. Diffusion of TetR proteins from one repressilator to its adjacent counterpart causes the same effect. This allows to control the frequency just by forcing using a sustained dissipation of diffusing TetR proteins.  Figure 2 illustrates a typical synchronisation run.

### 4.2. Synchronising Initial Phase Shifts

**Figure 3:**
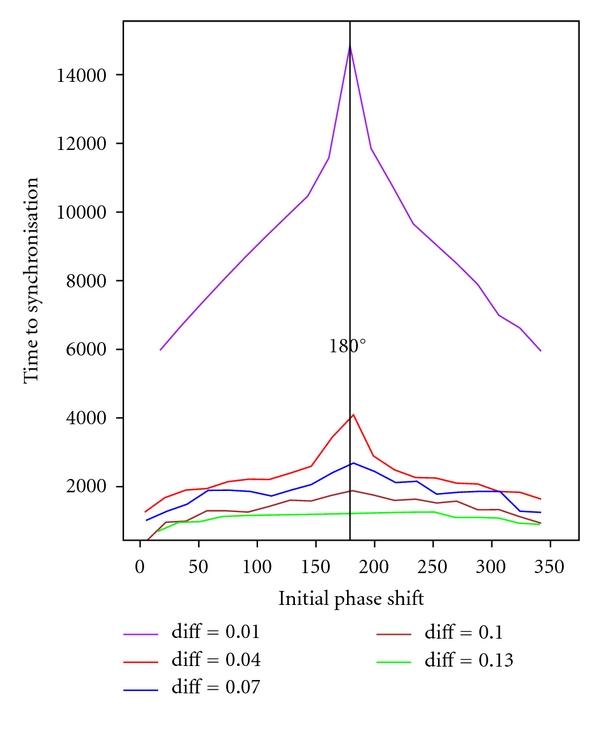
Time to synchronisation subject to various initial phase shifts. Parameter diff = 0.01,…, 0.13 denotes coupling strength from weak to strong coupling. Initial antiphase rhythmicity (phase shift 180°) between both repressilators causes the highest effort to synchronise both oscillatory signals by mutual forcing.

For the synchronisation study, we set up both repressilator's initial concentrations at the individual limit cycle in order to avoid effects occurring within the transient phase (stabilisation phase). Afterwards, a two-dimensional parameter scan was conducted varying the initial phase shift of both repressilators between 0° and 360° and simultaneously varying the coupling strength within the relevant range diff = 0.01 to 0.13 (weak to strong coupling). The time to synchronisation was obtained assuming a signal convergence of one minute per day (*ε*-neighbourhood's interval length = 1/1440 of oscillation period), see Figure 3.

The simulation study exhibits a correlation between coupling strength (diff) and time to synchronisation. Since a strong coupling (diff = 0.13) has a more significant effect on the adjacent repressilator's behaviour, synchronisation is achieved fast. In this case, even the influence of different initial phase shifts can be widely neglected. The situation becomes different when considering a weak coupling. Here, the initial phase shift predominantly determines the time to synchronisation. Initial antiphase rhythmicity (phase shift 180°) between both repressilators causes the highest effort to synchronise both oscillatory signals by mutual forcing. In this context, it is interesting to mention that the ability of the repressilator coupling to synchronise initial antiphase rhythmicity is a direct consequence of the (slight) asymmetric oscillatory signal shape. While symmetric oscillation curves (like sinusoidal signals) persist in antiphase when coupled, hence, unable to synchronise, asymmetric curves (like spiking signals) entail a kind of unbalanced response to forcing. There is no equilibrium between forcing effects shortening and those advancing the oscillatory period. The remaining effect is sufficient to initiate synchronisation. The slight asymmetry of the diagram in Figure 3 also results from the asymmetric shape of the repressilator's oscillatory signal. Interestingly, a medium coupling strength (diff = 0.07) generates a behaviour in which time to synchronisation for increasing initial phase shift can be compensated within a range of approximately 50°–100° and 260°–310°, respectively. 

### 4.3. Synchronisation of Different Initial Frequencies

We demonstrate the ability of the repressilator coupling to synchronise different initial frequencies in the elementary repressilators. To this end, individual protein degradation rates *k*_*lp*_, *k*_*tp*_, *k*_*cp*_ had been modified in conjunction with setting up all initial concentrations at the individual limit cycle. From this, we conducted a parameter scan taking into account the ratios of initial frequencies.

The purpose of this case study is to answer four questions. (1) Is there any synchronisation window, a continuous range of parameter settings, that runs the entire system into synchronisation? In other words, can we detect a variant of a so-called Arnold tongue? (2) If a synchronisation window could be identified, which of the three conditions necessary for synchronised oscillations become violated by leaving the delimiting parameter settings? (3) How is the time to synchronisation distributed within the synchronisation window? (4) Which synchronous frequency does result from the initially different frequencies after synchronisation?

While question (1) seems suitable to be answered in part using the Kuramoto method [3], an analytical ODE-based technique, a sufficient clarification of questions (2), (3), and (4) requires an explorative simulation study. An essential part of this study is the calculation of the frequencies governed by an oscillatory signal. To this end, we utilise the discrete fast Fourier transformation (FFT) for long-term data accompanied by sampling and counting of local oscillatory signals maxima or minima for short-time data series. Time to synchronisation is again measured by the number of elapsed time steps up to convergence of one minute per day (cf.  Section 4.2).

If synchronisation is obtained, we can distinguish two qualitative scenarios characterised by the resulting synchronous frequency in relation to either initial frequencies.

**Figure 4:**
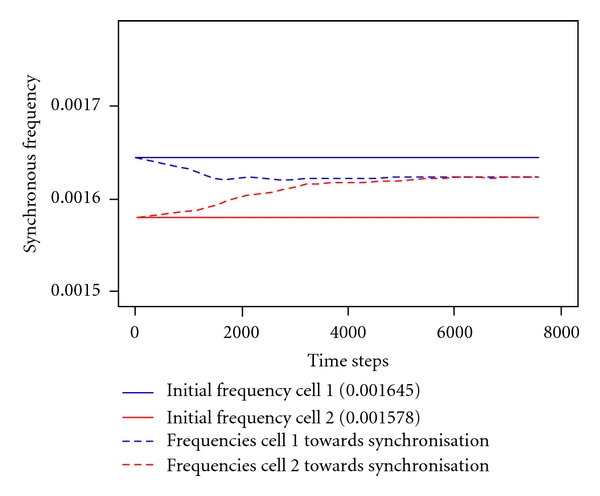
Typical temporal course towards synchronisation of two marginally different initial frequencies (solid lines) converging to a common value (dashed curves). Coupling strength: diff = 0.01, ratio of initial frequencies: 0.001645/0.001578 ≈ 1.042. Synchronous frequency: 0.001616.

Figure 4 depicts a typical temporal course towards synchronisation of two *marginally* different initial frequencies (solid lines). During the synchronisation process, both frequencies converge to a common value (dashed curves). This value deviates from both initial frequencies but arises in between. The synchronisation itself runs rather fast.

**Figure 5:**
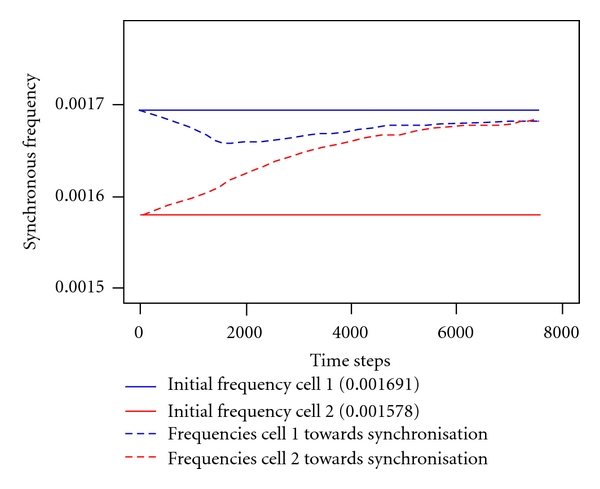
Typical temporal course towards synchronisation at the boundary of the synchronisation window. Synchronous frequency asymptotically reaches the maximum of either initial frequencies (dashed curves). Initial frequencies marked by solid lines. Coupling strength: diff = 0.01, ratio of initial frequencies: 0.001691/0.001578 ≈ 1.072. Synchronous frequency: 0.001690.

In contrast, a stronger—however *slight*—deviance of initial frequencies turns the synchronisation into a final frequency asymptotically converging to the maximum initial frequency, see Figure 5 for an example. Here, the synchronisation process takes more time.

The latter case coincides with arrival at the limits of the synchronisation window marking the maximal deviance of initial frequencies leading to synchronisation. Inside the synchronisation window, the synchronous frequency becomes adjusted in between of both initial frequencies, and the more we approach towards the boundaries of the synchronisation window, the synchronous frequency converges to the maximum of both initial frequencies.

**Figure 6:**
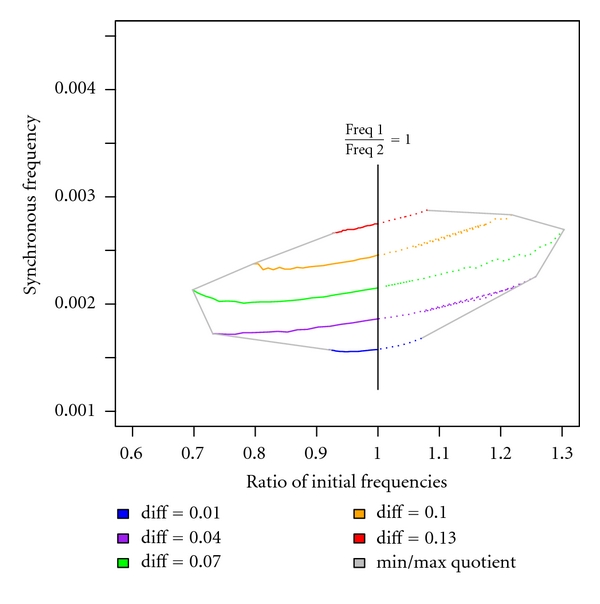
Synchronisation window: ratios of initial frequencies subject to synchronous frequency considering a variety of relevant coupling strengths diff = 0.01,…, 0.13 (variant of an Arnold tongue, a circle map disclosing dependencies of system parameters within a range of stable oscillation). Due to the bidirectionally balanced coupling strength, an almost symmetric synchronisation window can be obtained which is delimited by polyfrequential oscillations with respect to the ratios of initial frequencies and loss of undamped oscillation with respect to coupling strength.

We obtain a synchronisation window delimited by polyfrequential oscillations with respect to the ratios of initial frequencies and loss of undamped oscillation with respect to the coupling strength, see Figure 6. We checked whether an oscillatory signal is undamped or not by evaluating the eigenvalues of the Jacobian matrix derived from the ODEs specifying the reaction-diffusion kinetics.

Moreover, the simulation results indicate that a medium coupling strength (diff = 0.07) enables synchronisation within the largest ratio of initial frequencies ranging from 0.697 to 1.294. This means in terms of systems application for clock synchronisation that a clock signal can be temporarily slowen down (postpone the clock) and speeded up (put the clock forward) with up to approximately 30% of its velocity. The knowledge about parameterisation, capabilities, and limits of an oscillatory system envisioned to act as a biological clock is essential for subsequent integrative modelling, synthesis, and implementation of a corresponding frequency control system.

Bidirectionally coupled repressilators exhibit the ability to synchronise their oscillatory signals by forcing. It has been observed that arbitrary initial phase shifts become compensated while an adaption of the entire system to different initial frequencies of the single oscillators spans a synchronisation window. 

## 5. External Synchronisation: Repressilator as a Core Oscillator

**Figure 7:**
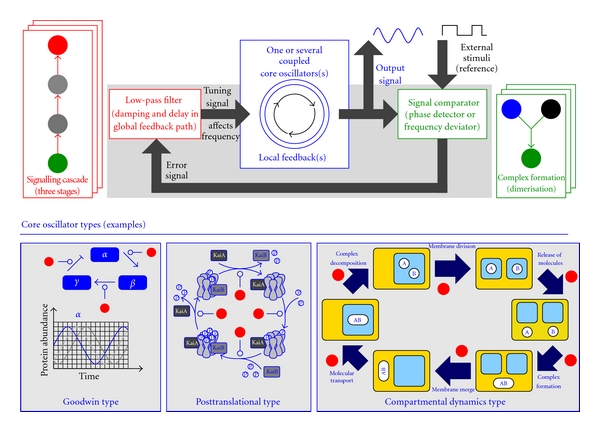
General scheme of a pure chemical frequency control system based on the concept of phase-locked loops (PLLs). The upper part shows the coupling structure of the three essential modules: core oscillator, signal comparator, and low-pass filter. Each module can be represented by numerous reaction networks. For instance, complex formation suffices for acting as signal comparator while a signalling cascade exemplifies a low-pass filter. Different types of core oscillators complete the control circuit.

The repressilator can be seen as an advantageous tool to conduct external synchronisation when embedded as core oscillator into a frequency control system based on the concept of phase-locked loop [23], PLL for short. These systems adapt their oscillatory output signal to an external stimulus acting as reference. In contrast to internal synchronisation, the external stimulus is not affected. A biological example is given by circadian clocks that harmonise their oscillatory behaviour with the daily light-dark rhythmicity [9]. Here, the light acts as external stimulus.  Figure 7 illustrates the general scheme of PLL. One or several coupled core oscillators constitute its central part. The signal comparator as downstream module determines the difference between core oscillator output and external stimulus. The phase shift between either signals is an ideal candidate to form an error signal able to adjust the core oscillator. The error signal passes a global feedback path along with damping and delay by dedicated low-pass filters. Finally, the resulting smoothened signal influences the core oscillator(s) by increasing or decreasing its frequency.

We expect to demonstrate that all functional modules required for a PLL control system can be implemented as interacting reaction networks. Both modules, signal comparator and global feedback path, efficiently employ low-pass filters. Signal transduction cascades found in cell signalling networks are a common biological motif to cover the functionality of low-pass filters [24]. Here, a focal protein alters its chemical state according to a trigger signal. A chemical state is specified by the addition or removal of phosphate groups to/from the focal protein. In case of low-frequency triggers, the subsequent modification of the chemical state can follow. Along with increasing frequency of the trigger, a threshold exists denoting that the reaction system is now too slow to follow the trigger and ends up in a steady state by means of a chemical equilibrium. Acting as a moving average element, the low-pass filter converts the output of the signal comparator into a delayed and damped error signal which is subsequently treated by the core oscillator in order to adjust its frequency. A linear pathway, typically between three and eight stages, gives the simplest example for a pure chemical low-pass filter. Its behaviour can be specified by a so-called Bode diagram which depicts the intensity of signal weakening subject to different frequencies. While low-frequency signals pass the filter, higher frequency oscillations become more and more diminished and hence eliminated. In addition, a low-pass filter can be configured in a way to provide almost sinusoidal signals by means of the fundamental oscillation term (first harmonic of Fourier series).

Having a chemical low-pass filter at hand, the functionality of the global feedback path is completely covered. The signal comparator benefits from low-pass filters to obtain the fundamental frequency of both signals, core oscillator output, and external stimulus. Then, the phase shift between both signals or the signal difference, respectively, can be extracted by performing arithmetic operations. Reaction networks for this task are effectively feasible assuming that substrate species concentrations encode operands while product species concentrations (in steady state) constitute the operational output [25]. For example, the set of two reactions *X*_1_ + *X*_2_ → *Y* and degradation *Y* → *Ø* in conjunction with mass-action kinetics conducts a multiplication of the form *Y* = *X*_1_(0)*X*_2_(0) with initial concentrations *X*_1_(0) and *X*_2_(0) as multipliers. Addition, nonnegative subtraction and division can be expressed in a similar way. Interestingly, a single complex formation (dimerisation) conducting a multiplication in a mathematical manner succeeds for estimation of the phase shift between sinusoidal signals due to elementary trigonometric laws whereas also more sophisticated mechanisms could be involved.

The core oscillator must be able to vary its frequency according to the error signal produced by the low-pass filter. There are numerous types of core oscillators found in living organisms' circadian clocks. From today's perspective, the majority of core oscillators reveals the *Goodwin type* [10], a cyclic gene regulatory network composed of mutual activating and inhibiting gene expressions. Repressilators as well as coupled repressilators fall into this category. The commonly most effective way to influence the frequency of those oscillators is the modification of the velocity of protein degradation reactions, for instance, accomplished by specific proteases released by the low-pass filter. Furthermore, core oscillators can be of *posttranslational type* [26] exploiting a cyclic scheme of protein phosphorylation and dephosphorylation in conjunction with complex formation and decomposition. Here, the involved catalysts affect the frequency. The third and most complicated type of core oscillators includes *compartmental dynamics* advantageously modelled using *membrane systems* combining representation of dynamical structures with tracing their spatiotemporal behaviour [27].

## 6. Conclusions

Bidirectionally coupled repressilators synchronise their oscillatory signals by forcing. Arbitrary initial phase shifts become compensated while adaptation to different initial frequencies spans a synchronisation window. Coupled repressilators can be seen as a part of a biological control system based on the concept of phase-locked loops. Further research has been directed to finalise the entire frequency control system by integration of additional components for signal comparison and damping, demonstrated by low-pass filters biologically implemented as specific signal transduction cascades. The simulations described in this paper were carried out using Copasi [28], statistical evaluation using [*R*].
